# Use of Alginate Extracted from Moroccan Brown Algae to Stimulate Natural Defense in Date Palm Roots

**DOI:** 10.3390/molecules25030720

**Published:** 2020-02-07

**Authors:** Soukaina Bouissil, Zainab El Alaoui-Talibi, Guillaume Pierre, Philippe Michaud, Cherkaoui El Modafar, Cedric Delattre

**Affiliations:** 1 Laboratoire de Biotechnologie et Bioingénierie Moléculaire, Faculté des Sciences et Techniques Marrakech, Université Cadi Ayyad, Marrakech 40000, Morocco; soukaina.BOUISSIL@etu.uca.fr (S.B.); zainab.elalaouitalibi@gmail.com (Z.E.A.-T.); elmodafar@uca.ac.ma (C.E.M.); 2 CNRS, SIGMA Clermont, Institut Pascal, Université Clermont Auvergne, F-63000 Clermont-Ferrand, France; guillaume.pierre@uca.fr (G.P.); cedric.delattre@uca.fr (C.D.); 3 Institut Universitaire de France (IUF), 1 Rue Descartes, 75005 Paris, France

**Keywords:** alginate, brown algae, date palm, naturel defense, phenolic metabolism, phenylalanine ammonia-lyase

## Abstract

Our study aimed to search for seaweed polysaccharides able to stimulate date palm defense mechanisms. Extraction, purification, characterization, and elicitor activity of sodium alginate (FSSA and BBSA) from Moroccan brown seaweeds *Fucus spiralis* and *Bifurcaria bifurcata* were investigated. FSSA and BBSA were characterized by proton nuclear magnetic resonance spectroscopy (^1^H-NMR) and size exclusion chromatography (HPLC-SEC). The mannuronic acid/guluronic acid (M/G) ratio of FSSA was M/G = 0.92 indicating that FSSA contained 48% and 52% of mannuronic and guluronic acids respectively, and the M/G ratio of BBSA was 0.47 indicating that BBSA contained 32% and 68% of mannuronic and guluronic acids respectively. Elicitor activity of FSSA and BBSA was carried out by developing an innovative study model on the date palm. The elicitor capacities were evaluated by investigating phenolic metabolism including phenylalanine ammonia-lyase (PAL) activity and total polyphenol content in seedling roots of date palm maintained in alginates solution (FSSA and BBSA) at different concentrations. The results obtained show that the PAL activity and the phenolic compound content were significantly stimulated with 1 mg·mL^−1^ of FSSA and BBSA; after 1 day of treatment with FSSA, and after 12 h of treatment with BBSA. These results show clearly those alginates extracted from Moroccan brown algae induced in date palm roots the stimulation of natural defense mechanisms.

## 1. Introduction

Recently, researchers have given more and more consideration to natural polysaccharides thanks to their huge properties such as stability, biodegradability, and biocompatibility for food and therapeutics applications. These considerable characteristics allow algal polysaccharides and their derivatives such as low molecular weight polymers and oligosaccharides structures to have great potential to be used in lots of domains such as agriculture (fertilizer, bio-elicitor, stimulators, signaling molecules, and activators). In the same way, the search for new strategies ensuring sustainable protection of crops at lower ecological cost is essential, given the environmental and health challenges related to the increased use of chemical pesticides, in order to meet these environmental and societal requirements in terms of phytoprotection, stimulating the natural defenses of plants represents a promising approach in terms of phytosanitary control [1]. Recent breakthroughs in molecular biology and plant transformation have shown that sensitizing a plant to respond more quickly to infection can give it increased protection against virulent microorganisms [2]. In parallel with this research, the demonstration of an induction of defense mechanisms following chemical and biological products has brought a new dimension to the concept of induced resistance [3]. We noted that the plant had the capacity to perceive and decode an external signal which, in turn, triggered a whole series of events leading to the coordinated synthesis and to the sequential accumulation of defense molecules.

Due to their effects as plant protectants, it has been proposed that algal polysaccharides act as elicitors of plant defense responses, to the extent that these algal polysaccharides have shown their effectiveness as protective agents against attacks by phytopathogens on tomatoes [4], post-harvest apple [5] and the olive tree [6]. It has been documented that purified polysaccharides from seaweed as well as oligosaccharides derived from them have the ability to trigger plant defense responses [7]. This is particularly the case for ulvane, laminarins, sulfated fucans, carrageenans, and alginates. The latter is an unbranched binary copolymers, consisting of (1->4) linked *β*-D-mannuronic acid (M) and α-L-guluronic acid (G) residues [8,9] organized as homo MM blocks and GG blocks and/or heteropolymeric MG/GM blocks [9] according to algae, where they are developed and the parts used for extraction [10]. Several studies have revealed its effectiveness in inducing defense mechanisms in plants notably, Madagascar periwinkle [11], grapevine [12], soybean [13], rice [14], and wheat [15]. However, the involvement of alginate in the elicitation of date palm defenses has never been reported. Therefore the objective of this work was to examine for the first timethe capacity of tow alginates extracted from *Bifurcaria bifurcata* R. Ross and *Fucus spiralis* L.brown algae in inducting date palm defense reactions, namely PAL activity as well as the accumulation of total phenolic compounds. 

## 2. Results

### 2.1. Extraction Yield and Chemical Composition of FSSA and BBSA

**Figure 1:**
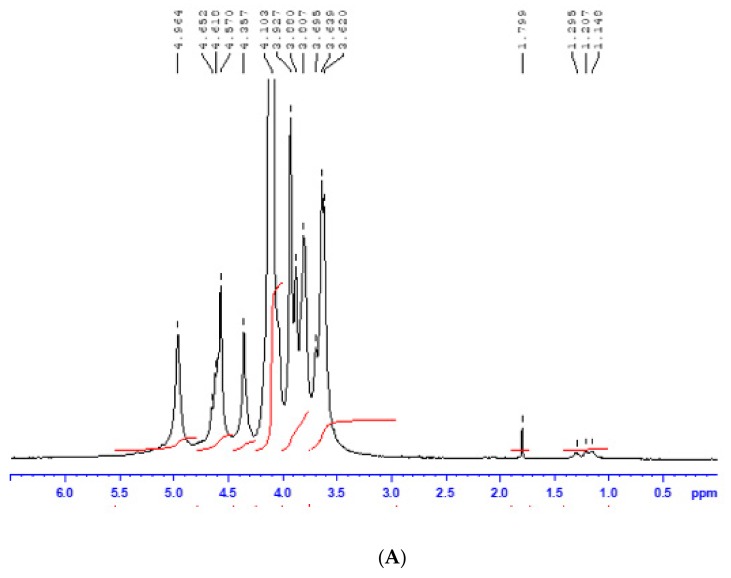

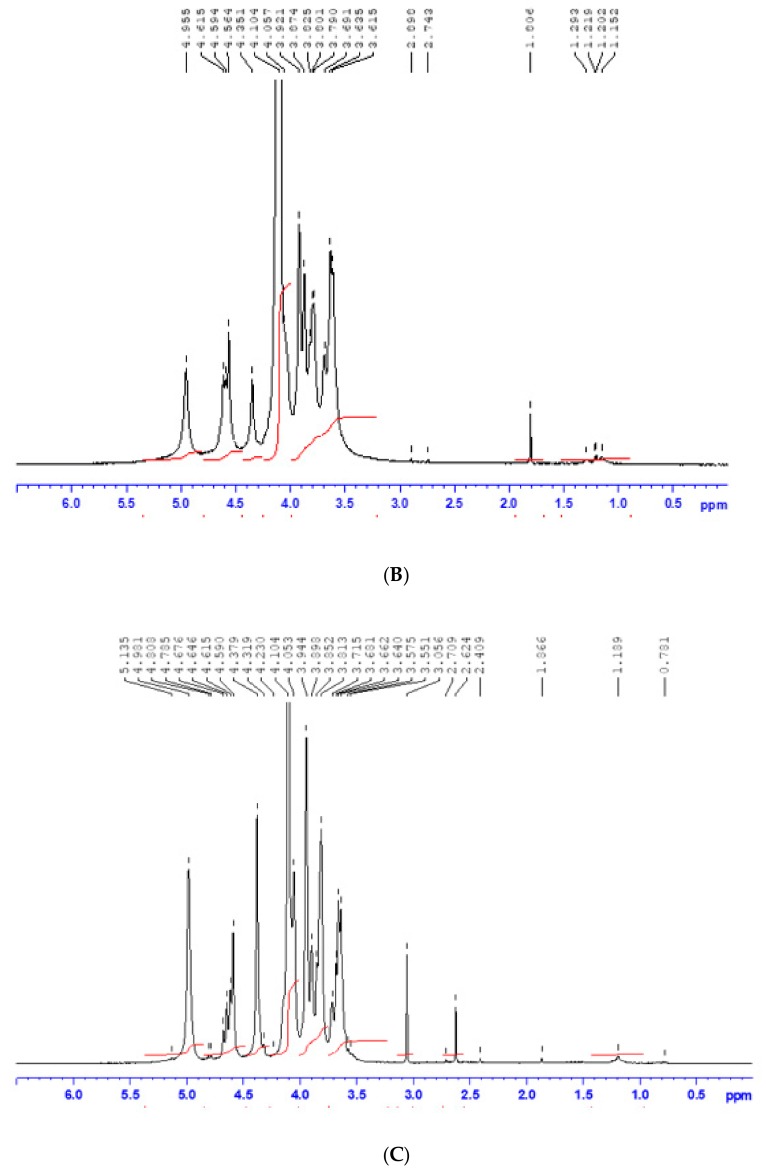
^1^H NMR spectra of FSSA (**A**), BBSA (**B**) and commercial alginate from Sigma (Ref: W201502-1KG) (**C**). The analysis was recorded at 60 °C for sample in D_2_O solution (20 g·L^−1^).

Purification of the aqueous alkaline extracts from *F. spiralis* and *B. bifurcata* gave sodium alginate samples in 25 and 24% yield respectively based on dry weight. The purified sodium alginates (FSSA and BBSA) yields were summarized in Table 1. The results of colorimetric assays reported in Table 1 show that alginate BBSA contained 48.6% of total sugars, including 58.4% of uronic acids and 18.25% of neutral sugars with minor amounts of polyphenols 6.44%. While FSSA contained 50.5% of total sugar principally uronic acids with 52.79%, and 16.12% of neutral sugars. Note to mention for each alginates the presence of low number of sulfates (<2% (*w/w*)) indicating low traces of fucoidans co-extracted from brown algae. It is also important to note that during the neutral sugars colorimetric assay, there are relative interferences between the neutral sugars and the acid sugars which leads to errors in quantification [16]. For more confirmation, we have also quantified the neutral sugars in commercial alginate (Sigma) and we have found that the commercialized alginate contained 15.61% (*w/w*) of neutral sugars with 81% of uronic acids. However, the ^1^H NMR spectrum of the same product (Figure 1C) does not reveal characteristic signals of neutral sugars (excepted the low signal around 1.189 ppm from fucose signal), which confirms the interferences of colorimetric assays. 

### 2.2. Structural Characterization of FSSA and BBSA by HPSEC and ^1^H-NMR

High-Performance Size Exclusion Chromatography (HPSEC) analysis was performed to evaluate the molecular weight of FSSA and BBSA. As mentioned in Table 2. BBSA was characterized by a weight average molecular weight (Mw) of 22 × 10^4^ g/mol. While average molecular weight of FSSA is 22.5 × 10^4^ g/mol. The ^1^H-NMR spectrum of alginates (Figure 1) revealed signals for BBSA (Figure 1B) and FSSA (Figure 1A) of the guluronic acid anomeric proton (G-1) at 4.995 and 4.964 ppm (signal I) respectively, and the overlap between the mannuronic acid anomeric proton (M-1) and the C-5 of alternating blocks (GM-5) at 4.594 and 4.618 ppm (signal II) while the guluronic acid H-5 (GG-5G) was identified at 4.351and 4.357 ppm (signal III) [17]. In addition, ^1^H-NMR analysis revealed also the characteristic signals of a fucose (≤0.2% (*w/w*)) at 1.295–1.148 ppm for FSSA, and at 1.293–1.152 ppm for BBSA, indicating the weak traces of fucoidans as neutral polysaccharides co-extracted from brown algae. All these NMR data are conformed to commercial alginate (Figure 1C) and reveal the high purity of our alginates from *Fucus spiralis* and *Bifurcaria bifurcata*.

Taking into account the Grasdalen [18] method described in Section 4.5, we have calculated frequencies of structural blocks of alginates (Table 2). The values of F_MM_, F_GG_, F_GM_ (or F_MG_) blocks and the M/G ratio were respectively evaluated at 0.09, 0.45, 0.23 and 0.47, indicating that BBSA was mainly composed of 68% guluronic acid and 32% mannuronic acid with abundance of homogenous doublet F_GG_. Whereas FSSA was composed of 52% guluronic acid and 48% mannuronic acid with equal amounts of homogenous doublet F_GG_ (0.37) and F_GM_ (0.33) and heterogenous blocks (F_MG_ and F_GM_) evaluated at 0.15. 

### 2.3. Effect of Brown Seaweed Alginates on Induction of the Phenolic Metabolism

#### 2.3.1. Phenylalanine Ammonia-Lyase (PAL) in Response to Elicitor Treatment

**Figure 2:**
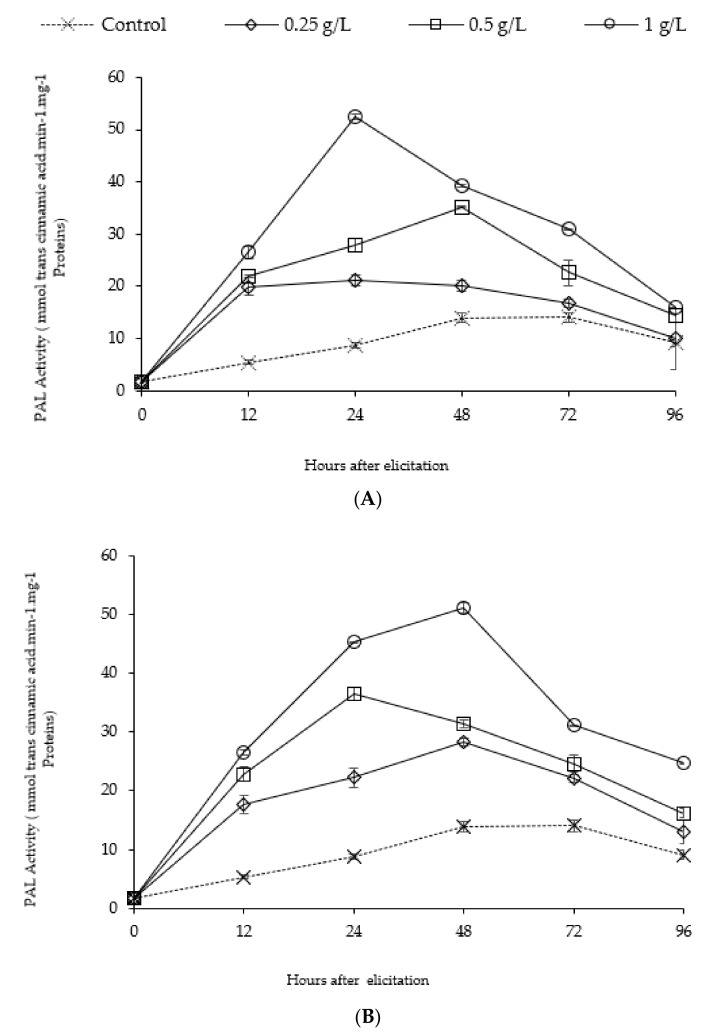
Time course induction of PAL activity of date palm roots in response of treatment by alginates of *F. spiralis* (**A**) and alginate of *B. bifurcata* (**B**).

Treatment of date palm by seaweed alginates increased the enzymatic activity of PAL in seedling roots. The application of the two seaweed alginates (BBSA and FSSA) caused differential response of the PAL activity (Figure 2). Elicitation by FSSA (Figure 2A), shows an early and significant induction of PAL activity compared to the control (*p* < 0.01). Indeed, a significant induction of the activity of this enzyme begins at 12 h in seedlings treated with FSSA, to reach its maximum at 24 h of treatment with 1 g·L^−1^ of FSSA. The significant induction of PAL by the 3 concentrations of FSSA was pronounced after 48h of elicitation. Nevertheless, treatment with 1 g·L^−1^ stands out significantly compared to other concentrations because of its intensity; 6 times higher than that observed in control plants at 24 h. However, the PAL activity in the roots treated by BBSA (Figure 2B) is precociously and significantly (*p* < 0.01) increased after 12 h of elicitation. This induction maintained in roots for 3 days after BBSA treatment with different concentrations. The treatment with 1 g·L^−1^ of BBSA at 12 h, 24 h, 48 h, and 72 h shows better performance in terms of induction of PAL 4.9; 5.16; 3.6 and 2.2 times respectively higher than that observed in control plants. Similarly, the efficiency of the concentration of 1 g·L^−1^seems better than that shown by 0.25 and 0.5 g·L^−1^ of both alginates (BBSA and FSSA). 

#### 2.3.2. Total Polyphenol Accumulation in Response to Elicitor Treatment

**Figure 3:**
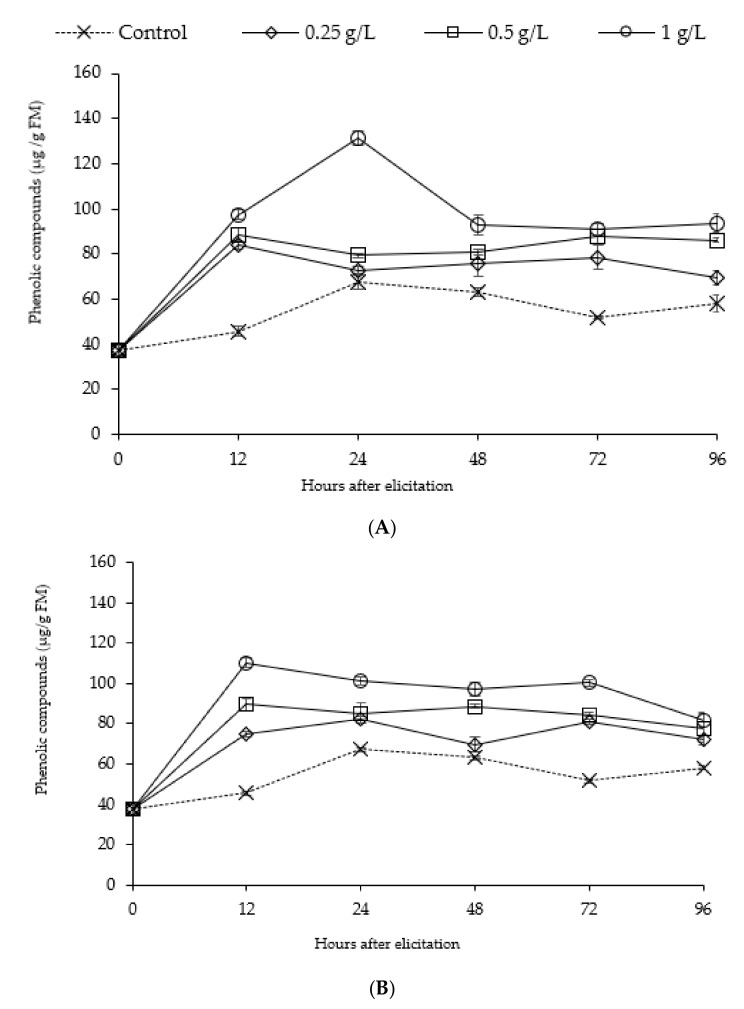
Phenolic compounds content accumulated in date palm roots after treatment by alginates of *F. spiralis* (**A**) and alginate of *B. bifurcata* (**B**).

Elicitation of date palm roots by alginates BBSA and FSSA occurred a significant (*p* < 0.01) and intense increase in total polyphenol content (Figure 3), of about 2.5 times higher than in the control from the first day of treatment by 1 g·L^−1^ of FSSA (Figure 3A). The polyphenol contents decreased subsequently from the second day for FSSA at 0.25, 0.5 and 1 g·L^−1^. Despite that, the values were significantly higher than in the control (*p* < 0.01). The highest accumulation was noticed in roots after 24 h of treatment by 1g·L^−1^of FSSA. Regarding the treatment by BBSA (Figure 3B), total polyphenol content increased precociously after 12 h of treatment by BBSA with all concentrations, this response maintained in roots for 3 days after BBSA treatment. Our results showed that the polyphenols accumulation followed almost the same kinetic regardless of the type of elicitor and its concentration. Indeed, the increase in concentration seems have a positive impact on phenolic metabolism, mainly in terms of the intensity of their accumulation.

## 3. Discussion

Extraction and characterization of alginates of two brown algae species harvested from the Moroccan coast was investigated as well as their elicitor capacity to induce natural defense in date palm roots. The content of total sodium alginates of *Fucus spiralis* L. (FSSA) and *Bifurcaria bifurcata* R. Ross (BBSA) were higer than that reported for *B. bifurcata* (16%) harvested from Britain [19], *F. visoculosis* (19%) [20] and *L. vadose* (3–17.6%) [15], but lower than those reported for alginates from the species *Ecklonia cava* (35–38%), *Laminaria digitata* (40%) [21] and *Durvillaea antarctica* (53%) [22]. Structural characterization of BBSA by ^1^H-NMR (Figure 1B) revealed a low M/G ratio (<1) correspond to higher amount of guluronic (G) than mannuronic acid blocks (M) with high proportions of GG blocks (Table 2). This ratio was lower than those reported for sodium alginates extracted from other brown algae such as *Cystoseira* species (0.59 < M/G < 1.46) [23,24,25]. It was also close to the M/G ratio noted for sodium alginates of *Cystoseira myrica* (0.45) and *Laminaria hyperborea* (0.41) [18,24]. However, FSSA (Figure 1A, Table 2) organized as homomannuronic/guluronic blocks than heteroplymeric blocks (MG and GM) given its M/G ratio close to 1 (0.96). Similar results have been reported in the literature concerning sodium alginates extracted from *F. vesiculosus* (M/G = 1.17) [26], *Laminaria* species like as *L. japonica* (M/G = 1.86) [27] and *L. digitata* of Moroccan coast (M/G = 1.12) [21]. Xiao et al. [28], reported that the main features for determining physicochemical properties of sodium alginate were mainly related to the homogeneous doublet (F_GG,_ F_MM_) and M/G ratio.

Stimulation of the natural defenses of plants based on the use of algae polysaccharides as one of the most promising alternative strategies for crop protection has been widely documented [7,29,30,31,32,33,34,35]. Above all, The metabolism of phenylpropanoids is a specific biosynthetic pathway for plants. It allows the synthesis of a large number of secondary metabolites involved in plant defenses. Trans-cinnamic acid is the first phenylpropane synthesized from phenylalanine via the activity of phenylalanine ammonia-lyase (PAL). PAL is, therefore, the enzyme constituting the main entry point into the phenylpropanoid biosynthesis pathway. This enzyme is also involved in the biosynthesis of p-coumaroyl CoA, a central compound from which various families of phenolic compounds will be synthesized [36]. Wherefore, the present work was particularly interested in the induction of PAL activity as well as the accumulation of phenolic compounds in the roots of date palm in response to treatment with alginates extracted from *B. bifurcata and F. spiralis*. These two defense indicators were chosen given their intense involvement in the defense mechanisms in date palm roots in response to an infection by *Fusarium oxysporium* f. sp albidinis [37]. The results obtained show that the metabolism of the phenylpropanoid pathway was inducible by alginates of the two studied algae. After 24 h, strong stimulation of PAL activity occurred in response to FSSA and BBSA treatment. The effectiveness of FSSA seems to be similar to that of BBSA. We noted also that, the induction of PAL activity was not closely correlated with increasing doses of the two alginates. Although PAL was clearly stimulated at doses of alginates (BBSA and FSSA) as low as 0.25 and 0.5 g·L^−1^, a dose greater than 0.5 g·L^−1^ was necessary to induce a detectable increase in PAL activity at 24 h of alginates treatment. This response is similar to that observed in wheat in response to 0.5 g·L^−1^of polymannuronic fraction of alginic acid [15], rice as a result of elicitation by 0.1 g·L^−1^ of oligoalginates [14], soybean cotyledon [13] and tabacco [38]. Different polysaccharides isolated from algae [4,5,39] have shown activities that elicit the defense mechanisms of various plants. Likewise, the stimulatory properties by various polysaccharides of marine algae, such as ulvan at 2 g·L^−1^ [6], oligoulvans at 5 g·L^−1^ [5], glucuronane at g·L^−1^ [4], carrageenane at g·L^−1^ [7,40] and with 5.4 µmol of oligocarrageenans [41] appeared to be similar to1 g·L^−1^ of *B. bifurcata* and *F. spiralis* alginates. It has been reported that the intensity of induction of this enzyme correlated with that of the induction of defense reactions by date palm in response to infection with the pathogen [42]. The sensitivity of certain varieties could therefore be the result of a weak induction of PAL activity [43]. This enzyme is considered to be the key enzyme in the metabolism of phenylpropanoids, leading to the biosynthesis of phytoalexins, fungitoxic phenolic compounds and lignin precursors [44,45,46,47]. 

The stimulation of PAL activity is accompanied by an increase in the content of phenolic compounds in date palm seedlings root pretreated with the two alginates. A similar profile noting an increase in PAL activity at 24 h and 12 h by 1 g·L^−1^ of FSSA and BBSA respectively were also obtained in case of phenolic compounds content in date palm seedlings root. We also notice that the polyphenols accumulation followed almost the same kinetic regardless of the type of elicitor and its concentration, while PAL activity showed different change trend after 24 h of algintes treatments. That can be explained by the possibility of polymerization of phenolic compounds in structural polymers, notably lignin and suberin deposited at the cell wall [48], as well as p-hydroxybenzoic, p-coumaric, ferulic, and sinapic acids [49]. The resistance of date palm cell walls to *Fusarium oxysporum* f. sp albedinis (Foa) hydrolytic enzymes is related to the intervention of those phenolic acids and lignin, which constitute a component of a mechanical defense [37]. In addition, the PAL activity not only governs synthesis of polyphenols, but also other pathways such as the synthesis of phytoalexins which are highly induced on date palm roots in response to infection by Foa [50]. 

Similary response noted in wheat in response to *Lessonia vadosa* alginate treatment [15], and in date palm root treated with 1 g·L^−1^ of chitosan [51]. This result is predictable since this enzyme catalyzes the conversion of L-phenylalanine to trans-cinnamic acid, a precursor of most phenolic compounds [52]. Their involvement in plant resistance is widely reported [47,53,54] and their role has been shown to be key in plant-pathogen interactions. It has been reported that the differential induction of defense mechanisms may be linked to a difference in the level of PAL activity between the different cultivars of the date palm, which is more effective and earlier in the resistant than in the susceptible [42,49,50,55,56]. Another work noted a higher accumulation of phenolic compunds in roots of two date palm cultivars, susceptible Jihel (JHL) and resistant Bousthami (BSTN) in response to SA treatment at 0.05 mM [57]. Phenolic compounds are also precursors of lignin whose role in defense is widely verified [4,5,42,58,59]. 

It is now well recognized that the bioactivity of saccharides could be linked to the difference in their physicochemical properties and subsequently, the signaling pathways activated in the plant. The latter can activate distinct defenses depending on the type of pathogen encountered or the elicitor applied [60]. This could be explained by the specificity of the membrane receptors recognizing the eliciting saccharide motifs and subsequently the induced signaling pathways [61]. Potential plasma membrane receptors and signaling pathways involved in the interaction of alginates and their derivatives with plant cells have not yet been identified [62]. Based on the present study, the ability of brown algal alginates BBSA and FSSA to enhance a wide range of defense responses in date palm seedlings roots might be owed to their uronic acids. Alginate appeared to have similar structure to polygalacturonic acid [10]. As shown in the case of galacturonic acid, Akimoto et al. [63] assumed that carboxylic groups have an important role in the initiation of the elicitation reaction in plant cells. In wheat, stimulation of PAL activity and the accumulation of phenolic compounds were greater following treatment with the polymannuronic fraction (Poly-M) than with the polyguluronic fraction (Poly-G) [15]. likewise Murphy et al. [64] reported that oligomannuronate as elicitors could enhance *bacitracin* A production in liquid cultures of *Bacillus licheniformis*. However and despite the difference between the alginates studied in the MM and GG blocks, there is no significant difference in elicitor effect (1.41% of difference in biological activity) between BBSA and FSSA, which can be linked to significant deviations, and not to the block MM and GG in each alginate. In the ranges of concentrations chosen (0.25 to 1 g·L^−1^) the behavior of BBSA and FSSA solutions was of a Newtonian fluid nature, despite the structural difference between them. It has been reported that the rheology of alginate of *Cystosiera humilis* harvested from the same station (EL jadida, Morocco), and of *Cystosiera compressa* harvested from Tunisia with M/G = 1.46, 0.77 and F_MM_= 0.40; 0.40 F_GG_= 0.21; 0.53 respectively [25,65] shows Newtonian behavior (water behavior) at concentrations greater than 2.5 g·L^−1^ [65] and 5 g·L^−1^ [25]. The shear-thinning behavior is an advantage for potential uses of alginates in the textile and food industries [66]. Further studies will be needed to better understand the effect of MM and GG blocks on elicitor activity in date palm seedlings roots. 

In summary, the results indicate that alginates of *B. bifurcata* and *F. spiralis* induce date palm natural defenses by enhancing PAL activity as well as phenolic compounds content which opened interesting prospects for firstly, examinating the capacity of BBSA and FSSA oligoalginates to induce date palm natural defenses. Studies will be done on the effect of oligoalginates with diverse Mw to see if we increase activity. It was foreseeable that the oligomers that exhibit a reduced viscosity and a low degree of polymerization exhibit better absorption at the root level and subsequently better efficiency in transducing the eliciting signal [61]. Secondly, for date palm crops protection against Bayoud desease. 

## 4. Materials and Methods 

### 4.1. Algae Material

*Bifurcaria bifurcata* R. Ross and *Fucus spiralis* L., two brown algae from the Atlantic coast of El Jadidacity Morocco, were harvested in December 2017 then washed, dried in the shade for 15 days and then in the oven at 50 °C ± 2 °C for 6 h. The dried samples were then ground into powder using a mechanical mixer and sifted with a mesh size of 0.5 mm.

### 4.2. Extraction and Purification of Sodium Alginates (BBSA, FSSA)

Alginate extraction from *Fucus spiralis* L. and *Bifurcaria bifurcate* R. Ross was carried out using high temperature alkaline extraction according to Davis et al. [67]. A total of 25 g of depigmented and dried algae were treated twice with 500 ml of HCl (0.1 M) for 2 h at 60 °C, pH 2.0 with constant stirring (250 rpm). After centrifugation (5000 rpm, 15 min, 4 °C), the residue (pellet) was washed with distilled water and then treated with Na_2_CO_3_ solution (3% *w/v*, pH 11.0) at 60 °C for 2 h with constant stirring (250 rpm). The supernatants were collected and precipitated with 3 volumes of ice-cold ethanol 96%. The precipitate recovered by centrifugation was suspended in distilled water and acidified with HCl (6M) to pH < 3.0 to precipitate alginic acid which was then resuspended in distilled water and neutralized at pH 7.5. The purification of alginates was performed by three successive precipitations with 3 volumes of ice-cold ethanol 96%. The final precipitate obtained was resuspended in distilled water and lyophilized to give *F. spiralis* sodium alginate (FSSA) and *B. Bifurcata* sodium alginate (BBSA) powders. 

### 4.3. Chemical Analysis of FSSA and BBSA

Uronic acids in FSSA and BBSA were quantified by using the procedure described by Blumenkrantz and Asboe-Hansen [68], with d-glucuronic acid as a standard. Neutral sugars were determined by using the sulfuric resorcinol method [69], with d-glucose as standards. Total sugar concentration was assayed by the colorimetric method of Dubois et al. [70], using the phenol and sulfuric acid assay. According to Bradford [71] method (Coomassie Brilliant Blue G-250 method), protein concentration was determined using bovine serum albumin (BSA) as standard. The sulfation degree was evaluated according to the turbidimetric method (BaCl_2_/gelatin) described by Dodgson and Price [72]. Total phenolic compounds content was quantified by the Folin–Ciocalteu method using Gallic acid as standard [73].

### 4.4. High-Performance Steric Exclusion Chromatography (HPSEC) Analysis

Molecular weight analysis of extracted polysaccharides was performed by Size Exclusion Chromatography (SEC) on an Agilent 1100 Series high-performance liquid chromatograph (Agilent Technologies, Santa Clara, CA, U.S.A.). The device has a RID (Refractive Index Detector) cell to detect substances with little or no uptake in the UV (including polysaccharides) through the variation of refractive index relative to the buffer. The separation was carried out in two columns: TSK G5000PWXL and TSK G3000PWXL (Tosoh Bioscience, Tokyo, Japan) coupled in series. Isocratic elution with sodium nitrate (NaNO_3_, 0.1 M) was applied and the flow rate was maintained at 1 mL·min^−1^. A standard range of Pullulan (Sigma–Aldrich, St. Louis, MO, U.S.A.) of different molar masses was used. The standards were dissolved at 10 g·L^−1^ in NaNO_3_ buffer (0.1 M) and then filtered through 0.22 μm (Sartorius Ministart RC4). The samples are dissolved at 10 g·L^−1^ in NaNO_3_ buffer (0.1 M) and then filtered through 0.45 μm.

### 4.5. ^1^H NMR Spectroscopy Analysis

Alginates FSSA and BBSA were dissolved at 20 g·L^−1^ in D_2_O (99.9% D) three times and lyophilized. Before analysis, samples were then dissolved in D_2_O (20 g·L^−1^). ^1^H NMR spectra was recorded at 60 °C using a 400 MHz Bruker Avance spectrometer (Bruker Scientific Instruments, USA) equipped with a BBFO probe. The ^1^H NMR experiments were applied with a spectral width of 3000 Hz. The analyses were compared by using a commercial sodium alginate (Sigma, Ref: W201502-1KG). The individual blocks of guluronic and mannuronic acids (F_G_ and F_M_), the homogeneous (F_GG_ and F_MM_) and heterogeneous (F_GM_ and F_MG_) blocks of FSSA and BBSA were calculated using the areas of (I, II and III) signals according to Grasdalen [18] method and the Equations (1)–(3):

(1) $$F_{G}=\frac{A_{I}}{A_{II}+A_{III}}$$

(2) $$F_{GG}=\frac{A_{III}}{A_{II}+A_{III}}$$

(3) $$F_{M}=1-\;F_{G}$$

The double fractions (F_GM_ and F_MM_) and M/G ration of alginates were deduced by referring to the Equations (4)–(6):

(4) $$F_{GM}=F_{MG}=F_{G}-\;F_{GG}$$

(5) $$F_{MM}=F_{M}-\;F_{MG}$$

(6) $$\frac{M}{G}=\frac{1-F_{G}}{FG}=\frac{F_{M}}{F_{G}}$$

### 4.6. Plant Material

Disinfected seeds of Jihel, a sensitive variety of date palm (*Phoenix dactylifera* L.) were germinated in sterile sand (180 °C for 3 h) at 38 °C for 3 weeks. Germinated seeds are then transferred to hole-based alveoli containing substrate composed of sand, soil and potting soil (1v/1v/1v). The culture is maintained under a greenhouse at 30 °C and under a photoperiod of 16 h/8 h (day/night), until the leaf and a half stage (2 months). 

### 4.7. Elicitation Test 

After 2 months of culture, the roots of date palm seedlings are soaked in aqueous solutions of alginates (BBSA and FSSA) titrated at a concentration of 0.25, 0.5 and 1 g·L^−1^ at pH 6.5. The control plants were treated with distilled water. The seedlings are then placed in a culture room at a temperature of 30 °C with a photoperiod of 16 h/8 h (day /night) and 240 μmol·(m^2^)^−1^·s^−1^ of the illumination intensity. The response to the elicitation was followed in the roots of seedlings during 96 h. For each treatment, the roots are removed, crushed into powder in liquid nitrogen and divided into 3 test portions for each biochemical assay: (1) phenylalanine ammonialyase (PAL) activity and (2) phenolic compounds content in roots. The reported data were the means of three repetitions with three seedlings per repetition. 

### 4.8. Extraction and Determination of Phenylalanine Ammonialyse (PAL) Activity

The elicitor capacity of *B. bifurcata* and *F. spiralis* alginates was evaluated by analyzing the PAL activity according to the method described by Liu et al., [74] with slight modifications. 250 mg of the root of the seedlings were ground in 3 mL of the ice-cold borate buffer (100 mM, pH 8.8) containing 1 mM EDTA and 5% insoluble polyvinyl polypyrrolidone (PVPP) (m/m). The homogenate was centrifuged at 10,000× *g* for 30 min. The supernatant recovered constitutes the enzyme extract. The extraction was performed at 4 °C. The reaction mixture consisted of 600 µL of the enzymatic extract, 250 μL of *L*-phenylalanine (20 mM) and 1 ml of borate buffer (100 mM, pH 8.8). After incubation for 1 h at room temperature 30 °C, the reaction was stopped by addition of 100 μL of HCl (6N). The PAL activity is determined by measuring the optical density (OD) at 290 nm. The increase in absorbance reflects the appearance of trans-cinnamic acid by conversion of *L*-phenylalanine under the action of PAL. In the same way, a curve standard was carried out with the cinnamic acid under the same experimental conditions. The PAL activity was expressed in mmoles of transcinnamic acid·min^−1^·mg^−1^ protein. 

### 4.9. Determination of Total Protein

The protein content was determined according to Bradford method [71]. 100 µL of the protein extract was added to 2.5 mL of Bradford reagent. The mixture was stirred than incubated for 5 min at 30 °C. The absorbance measured at 595 nm. The protein content of samples was determined by reference to a standard range of bovine serum albumin (BSA).

### 4.10. Extraction, Purification, and Determination of Total Polyphenol Content

The extraction of the phenolic compounds was carried out according to the method of Hagen et al. [75] with minor modifications, 500 mg of the root tissues were homogenized with 3 mL of ice-cold methanol 80% (*v/v*). The homogenate was then placed in an ultrasonic bath for 30 min and then centrifuged twice at 10,000× *g* for 15 min at 4 °C to exhaust the content of phenolic compounds. The supernatants recovered constitute the hydromethanolic phenolic extract. The purification was performed according to the following protocol; 1 mL of distilled water and ½ v of petroleum ether were added to the aqueous extract obtained after methanol evaporation (60 °C), to allow depigmentation and delipidation. After removal of the organic phase, 50 μL of metaphosphoric acid 2% (*v/v*) and ethyl acetate (*v/v*) were added to the aqueous extract. The ethyl acetate phase was then recovered and evaporated to dryness. The phenolic residue was dissolved in methanol 80% (*v/v*)for total phenolic determination according to the Folin–Ciocalteu method [76] 50 µL of the purified phenolic extract were added to 250 μL Folin–Ciocalteu reagent diluted 1/3, after stirring for 3 min, 500 μL of sodium carbonate 20% (*w/v*) was added. The mixture was incubated at 40 °C for 30 min. The absorbance values were read at 760 nm. The content of soluble phenolics was expressed by reference to a standard range made with gallic acid and reported in mg gallic acid·g^−1^ fresh matter (FM). 

### 4.11. Statistical Analysis

Data were expressed as mean and standard deviation. PAL and polyphenol contents results were tested by one-way analysis of variance (ANOVA) using Tukey test at *p* < 0.05 for multiple comparisons. All statistical analyses were performed using SPSS software Version 10.0.

## Figures and Tables

**Table 1:** Yield and chemical analysis of *Fucus spiralis* L. **(**FSSA) and *Bifurcaria bifurcata* R. Ross **(**BBSA) extracted from brown seaweeds.

Simples	Extraction Yield (% *w/w*)	Total Sugar (% *w/w*)	Uronic Acids (% *w/w*)	Neutral Sugar (% *w/w*)	Sulfates (% *w/w*)	Proteins (% *w/w*)	Phenolic Compounds (% *w/w*)
FSSA	25 ± 0.21	50.46 ± 0.35	52.79 ± 0.15	16.12 ± 0.71	1.91 ± 0.15	Traces *	Traces *
BBSA	24 ± 0.12	48.61 ± 0.45	58.44 ± 0.55	18.25 ± 0.85	1.78 ± 0.23	Traces *	6.44 ± 0.02

* Traces: % *w/w* ≤ 1.10^−3^.

**Table 2:** Structural characterization of FSSA and BBSA extracted from brown seaweeds.

Samples	Mw^a^(g/mol)	Frequencies of Structural Blocks ^b^						
		F_M_	F_G_	M/G	F_MM_	F_GG_	F_MG_	F_GM_
BBSA	22 × 10^4^	0.32	0.68	0.47	0.09	0.45	0.23	0.23
FSSA	22.5 × 10^4^	0.48	0.52	0.92	0.33	0.37	0.15	0.15

^a^ Mw: weight average molecular weight was measured by HPSEC. ^b^ M/G ratio and structural blocks was measured by ^1^H-NMR.
